# High Correlation of the Decayed, Missing, and Filled Teeth (DMFT) Index With Caries Experience in First Permanent Molars: Perspectives and Implications in Oral Epidemiology From a Cross-Sectional Study

**DOI:** 10.7759/cureus.74196

**Published:** 2024-11-22

**Authors:** Sandra I Jimenez-Gayosso, Norma L Robles-Bermeo, Rogelio J Scougall-Vilchis, Mariana Mora-Acosta, Juan A Casanova-Sarmiento, Horacio Islas-Granillo, Saraí C Guadarrama-Reyes, Raúl Argüello-Sánchez, Carlo E Medina-Solís, Taurino Amílcar Sosa-Velasco

**Affiliations:** 1 Dentistry, Academic Area of Dentistry of Health Sciences Institute, Autonomous University of Hidalgo State, Pachuca, MEX; 2 Dentistry, Advanced Studies and Research Center in Dentistry "Dr. Keisaburo Miyata" School of Dentistry, Autonomous University of State of Mexico, Toluca, MEX; 3 Dentistry, School of Dentistry, Autonomous University of Campeche, Campeche, MEX; 4 Dentistry, School of Dentistry, Autonomous University of the State of Mexico, Toluca, MEX; 5 Dentistry, School of Dentistry, Autonomous University “Benito Juarez” of Oaxaca, Oaxaca, MEX

**Keywords:** caries index, dental caries, first permanent molars, mexico, oral health

## Abstract

Background

Dental caries is identified as one of the most prevalent chronic pathologies among the pediatric population on a global scale, constituting a public health problem. Within the permanent dentition, the first molars play a fundamental and critical role both in masticatory functionality and in the development of occlusion and the overall oral health of the patient. Previous research has shown that permanent molars tend to show significantly high levels of caries incidence, and a correlation has been detected between the status of the first permanent molars and the overall caries rate.

Objective

To investigate the correlation between the overall DMFT index (Decayed, Missing, and Filled Teeth Index in the permanent dentition) and the DMFT calculated from the status of the first permanent molars (FPMs) in a sample of Mexican school-aged children, and to discuss the implications for oral epidemiology.

Material and methods

A cross-sectional study was conducted on 107 children aged 5 to 12 years at a pediatric dentistry clinic of a public university in Mexico. All data were obtained from medical records. The DMFT index was calculated for the entire dentition, and a separate DMFT index was determined for the first permanent molars. Nonparametric tests were used in the analyses. Analyses were performed in Stata 14 (StataCorp, College Station, USA).

Results

The mean age was 8.52±1.36 years, and 50.5% were boys. The mean overall DMFT index was 1.90±2.01 and the DMFT-FPM index was 1.71±1.74, a difference of 0.19 (10%) (Wilcoxon: DMFT vs DMFT-FPM, p=0.0009). The correlation between caries indices was very strong (Spearman r=0.9803; p<0.0001). The overall caries prevalence was 58.9% for DMFT>0 and for the DMFT-FPM>0, 57.0%, a difference of 1.9 percentage points (3.2%). The results show an association of experience and caries prevalence by age (p<0.05), but not by sex (p>0.05).

Conclusions

There is a very strong correlation between the overall DMFT index and the DMFT calculated exclusively for the first permanent molars in this sample of Mexican schoolchildren. This suggests that the FPMs contribute significantly to the overall caries experience and could be a key indicator in the diagnosis and monitoring of oral health, taking into account the percentage of underestimation.

## Introduction

Dental caries is one of the most prevalent chronic diseases among the pediatric population worldwide, representing a serious public health problem [1,2]. The World Health Organization estimates that caries affects approximately 60-90% of school-aged children, with countries in Asia and Latin America being the most affected, leading to a high demand for dental services and significantly impacting the quality of life of children and their families [3-6]. It is estimated that around 3.5 billion people are affected by disorders related to the mouth, of which 2.3 billion have untreated caries in their permanent dentition, and 532 million have untreated caries in their primary teeth [7]. Mexico is a country with a constantly growing population and demographic changes, where, despite advances in access to health services and the promotion of oral hygiene practices, oral diseases, such as caries, persist as a significant challenge across all regions of the country [8-11]. Dental caries affects a large proportion of the child, youth, and adult population, with a notably high burden in those sectors facing greater socioeconomic inequalities [2,10-14]. In terms of prevalence, it has been reported in Mexico that approximately 70% to 85% of 12-year-old children show evidence of caries in their permanent dentition, while 50% of 6-year-old children have caries in their primary dentition [15]. However, subsequent estimates have indicated a prevalence ranging from 30.7% to 79.2%, with an average DMFT (Decayed, Missing, and Filled Teeth) Index between 0.52 and 3.67. Similarly, estimates for 6-year-old children have shown a prevalence ranging from 26.3% to 77.5%, and a mean DMFT index between 0.73 and 5.35 [11]. In this context, the Decayed, Missing, and Filled Teeth Index is used as a crucial epidemiological tool to accurately quantify the prevalence of caries in permanent dentition. This allows for a comprehensive assessment of oral health status and dental care needs across various age segments [16-18].

In permanent dentition, the first molars play a crucial and essential role in both masticatory functionality and the development of occlusion and overall oral health of the individual. These teeth are critical for the proper alignment of the dentition and the optimal distribution of forces during the masticatory process, significantly contributing to the preservation of oral health throughout life [19,20]. The eruption of the first permanent molar marks the beginning of the mixed dentition, which is one of the most dynamic periods in the development and growth of the stomatognathic system, occurring at an early stage of life, around 6 to 7 years of age, exposing them for an extended period to the risk of developing caries, in contrast to other permanent teeth present in the oral cavity [21-25]. Additionally, it is crucial to consider that their morphology and position within the dental structure make them susceptible to the accumulation of plaque and food debris, significantly increasing their propensity for caries formation. As a result, many children need to visit the dentist and often require restoration or extraction of the first permanent molars (FPMs) [25,26]. Previous research has indicated that first permanent molars tend to exhibit considerably high levels of caries incidence in school-aged children, significantly contributing to the overall DMFT index in this demographic group. Therefore, estimating dental caries in the FPMs at both individual and community levels could help understand the pattern and severity of dental caries [16,27,28].

Previous studies have found a correlation between the status of the first permanent molars and the overall caries index [27]. This suggests that the status of the FPMs is a strong indicator of the overall oral health in a population. Understanding this correlation will help identify specific patterns of involvement, guide preventive programs, and design treatment strategies focused on preserving the first molars and reducing the burden of caries in the child population. This study aimed to test the following hypothesis: There is a significant positive correlation between the overall DMFT index and the DMFT of the first permanent molars in children, indicating that a higher degree of general caries is associated with a higher rate of caries in these specific teeth. The research question of the study was: Can DMFT-FPM serve as a reliable indicator of overall caries experience? Therefore, the objective was to investigate the correlation between the overall DMFT index and the DMFT calculated from the status of the FPMs in a sample of Mexican school-aged children and to discuss the implications in epidemiology within the global scenario of children's oral health.

## Materials and methods

Study design, population, and sample

A cross-sectional study was conducted on children aged 5 to 12 years at the Pediatric Dentistry Specialty Clinic of the Center for Research and Advanced Studies of the Autonomous University of the State of Mexico (UAEM CIEAO), which provides low-cost pediatric specialty services to the general population, with most patients from low to medium socioeconomic status. This report is part of a project that measured multiple indicators of oral health. The methodology regarding data on dental caries, dental pain, and dental trauma has been previously described [29-31].

A sample size calculation was performed using the G*Power program (version 3.1.9.7; Heinrich Heine University, Dusseldorf, Germany) [32,33]. The parameters entered into the analysis were: one-tailed, a medium effect size (f = 0.30), a significance level of 0.05, and a statistical power of 90, resulting in a sample size of 102. Additionally, a possible 5% loss due to attrition was adjusted for. This ensures a high probability of detecting significant differences between groups, minimizing the risk of Type II errors (false negatives). This approach allows for balancing the statistical validity of the study with the practical feasibility of managing an adequate sample size, providing robust and clinically relevant results [34]. Sample size calculation incorporates several key considerations: A one-tailed test was selected to enhance statistical power, given the study's directional hypothesis predicting a positive correlation between the variables. A medium effect size (f = 0.30) was chosen as a conservative estimate, representing a balance between statistical power and practical feasibility. This lines up with the GPower program's options, which include small (0.10), medium (0.30), and large (0.50) effect sizes. Furthermore, a power level of 90% (β = 0.10) was set to significantly minimize the risk of a Type II error, enhancing the study's reliability. Finally, the calculated sample size included an adjustment for potential participant attrition (5%), accounting for possible dropouts during the study.

The inclusion criteria for this analysis were: a) having at least one erupted first permanent molar that could be evaluated, b) both sexes; c) aged 5 to 12 years; d) children living in the community (i.e., non-institutionalized). The exclusion criteria were: a) medical records with confusing data or with information that had not been properly collected (for example, illegible or incomplete entries: handwritten notes may be difficult to decipher, or key information may be missing (e.g., specific teeth affected, treatment dates); inconsistent coding or terminology (different dentists may use different notations or abbreviations, making it difficult to standardize data for analysis); missing exams (a child may have had only a partial dental exam, meaning information about some teeth may be missing); and ambiguous descriptions (descriptions of caries may not be accurate or standardized, making it difficult to classify the severity or type of cavities consistently across all records)), and b) parents who did not consent to the taking of clinical photographs. The final sample consisted of 107 patient records.

Data and variables

All data were obtained from medical records previously completed by the parents and/or guardians of the patients. The dependent variables were the experience and prevalence of dental caries. The DMFT index (Decayed, Missing, and Filled Teeth in permanent dentition) was calculated; these indices determine the current and past experience of caries through their components [35]. The indices were obtained from the diagnostic records in the medical histories made by the specialty students under the supervision of their professors, which were then cross-checked against clinical photographs. The clinical records are reviewed by the clinic's professors.

In the original study, a series of sociodemographic and health-related socioeconomic variables were collected, which were not included in this analysis as they were not relevant to the purpose of the study. However, the variable for dental pain experience (0 = Never, 1 = Previously, 2 = Currently) was included to observe the behavior of both indicators of caries experience and prevalence.

Statistical analysis

Data analysis was performed using the Stata 14.0 statistical package (StataCorp, College Station, USA). In the univariate analysis, frequencies and percentages for qualitative variables, as well as means and standard deviations for quantitative variables, were reported.

In the bivariate analysis, non-parametric statistical tests were performed: Spearman, Mann-Whitney, and Chi-square tests. To contrast the DMFT (overall Decayed, Missing, and Filled Teeth index) and the DMFT-FPMs (Decayed, Missing, and Filled Teeth index in first permanent molars), the Wilcoxon signed-rank test was used. Nonparametric tests were chosen because the data did not meet the assumptions of parametric tests. Parametric tests require that the data be normally distributed and have homogeneity of variance. Caries data, such as DMFT scores, were not normally distributed and/or the variances differed significantly between groups (e.g., age groups or sex). Nonparametric tests are distribution-free, meaning they make no assumptions about the underlying distribution of the data.

Ethical considerations

This study was conducted following the guidelines for health research involving human participants in Mexico and the Declaration of Helsinki. Parents/guardians approved the use of the data for research purposes. The protocol was approved by the School of Dentistry of the Autonomous University of the State of Mexico (CIEAO/16/05).

## Results

The study included a total of 107 individuals, with descriptive results shown in Table 1. The average age was 8.52 ± 1.36 years, with 50.5% being boys. The overall average DMFT index was 1.90 ± 2.01, while the DMFT index for the first permanent molars was 1.71 ± 1.74, a difference of 0.19 (10%). In the Wilcoxon signed-rank test, the difference between the two indices was statistically significant (DMFT vs. DMFT-FPMs, p = 0.0009). The correlation between the two caries indices was very strong (Spearman r = 0.9803; p < 0.0001). The prevalence of caries calculated for the overall DMFT was 58.9%, while for the DMFT-FPMs, it was 57.0%, a difference of 1.9 percentage points (3.2%).

**Table 1:** Descriptive Analysis of the Study Sample. *Wilcoxon signed-rank test (DMFT vs. DMFT-FPMs): z = 3.315; p = 0.0009. Spearman correlation: r = 0.9803; p < 0.0001. SD: Standard Deviation. DMFT: Overall Decayed, Missing, and Filled Teeth index. DMFT-FPMs: Decayed, Missing, and Filled Teeth index in first permanent molars.

Variables	Average	SD
Age (years)	8.52	1.36
DMFT mean	1.90	2.01*
DMFT-FPMs mean	1.71	1.74*
	Frequency	Percentage
Sex		
Male	54	50.5
Female	53	49.5
Dental Pain		
Never	55	51.4
Previously	37	34.6
Currently	15	14.0
Caries Prevalence (%DMFT > 0)		
No caries	44	41.1
With Caries	63	58.9
Caries Prevalence on FPMs (%DMFT-FPMs > 0)		
No caries	46	43.0
With Caries	61	57.0

Table 2 presents the results of the bivariate analysis of caries experience (average) for both indices calculated by age and sex. The results show a significant correlation of the overall caries index (r = 0.5209; p < 0.0001) with the caries index in the first permanent molars (r = 0.5279; p < 0.0001) with age. On the other hand, when analyzing the indices by sex (Mann- Whitney test), no statistically significant differences were observed (p > 0.05). When analyzing both indicators of dental caries experience across the categories of the dental pain variable, significant differences were observed using the Kruskal-Wallis test, both in the overall DMFT (p < 0.05) and in the DMFT-FPMs, with lower averages in those who have never experienced dental pain, followed by those who had previous dental pain and then those with current dental pain.

**Table 2:** Distribution of DMFT and DMFT-FPMs Indices by Age and Sex. DMFT: Overall Decayed, Missing, and Filled Teeth index. DMFT-FPMs: Decayed, Missing, and Filled Teeth index in first permanent molars.

Variables	DMFT	DMFT-FPMs
Age	1.90±2.01	1.71±1.74
Spearman's rho	r = 0.5209	r = 0.5279
p-value	p < 0.0001	p < 0.0000
Sex		
Male	2.01±2.05	1.83±1.73
Female	1.79±1.99	1.60±1.75
Mann-Whitney	z = 0.432	z = 0.507
p-value	p = 0.6655	p = 0.6120
Dental pain		
Never	1.40±1.78	1.29±1.66
Previously	2.40±2.11	2.13±1.70
Currently	2.53±2.23	2.26±1.83
Kruskall-Wallis	X^2^ = 13.067	X^2^ = 6.752
p-value	0.0420	0.0342

Table 3 presents the results of the bivariate analysis of caries prevalence (percentage of affected individuals) for both indices calculated by age and sex. The results show, in the Mann-Whitney test, that the average age was higher in children with caries than in children without caries, both in the overall index (7.74±1.20 vs. 9.06±1.20; p < 0.0001) and in the caries index of the first permanent molars (7.75±1.17 vs. 9.10±1.20; p < 0.0001). On the other hand, when analyzing the prevalence of caries by sex (chi-square test), no statistically significant differences were observed (p > 0.05). Regarding the prevalence calculated for both indicators of dental caries across the categories of the dental pain variable, significant differences were observed (p < 0.05) when applying the chi-square test for DMFT-FPMs, although not (p = 0.099) for the estimated prevalence in the first permanent molars. 

**Table 3:** Distribution of Caries Prevalence (DMFT-FPMs > 0) by Age and Sex. DMFT: Overall Decayed, Missing, and Filled Teeth index. DMFT-FPMs: Decayed, Missing, and Filled Teeth index in first permanent molars.

Variables	DMFT > 0	DMFT-FPMs > 0
Age	No caries = 7.74±1.20	No caries = 7.75±1.17
	With caries = 9.06±1.20	With caries = 9.10± 1.20
Mann-Whitney	z = -5.127	z = -5.319
p-value	p < 0.0001	p < 0.0001
Sex		
Male	59.3%	59.3%
Female	58.5%	54.7%
Chi-square	X^2^ = 0.0065	X^2^ = 0.2252
p-value	0.936	0.635
Dental pain		
Never	49.1%	45.4%
Previously	67.6%	67.6%
Currently	73.3%	73.3%
Chi-square	X^2^ = 4.6244	X^2^ = 6.3100
p-value	0.099	0.043

In a complementary analysis (not presented in the manuscript), a negative binomial regression model was performed, which was adjusted for several variables such as: dental pain, age, sex, family size, frequency of tooth brushing, receiving help with tooth brushing and having preventive dental consultation, we obtained that, for each increase of one unit in the number of first molars with caries, the expected average of the DMFT index increases by a factor of 2.08. That is, the expected average of the DMFT index is expected to be 2.08 times higher in individuals with one permanent first molar with caries, compared to individuals with one unit less of carious first molars, keeping the other variables of the model constant.

## Discussion

This study primarily aimed to investigate the correlation between the overall DMFT index and the DMFT index calculated solely from the status of the FPMs in a sample of Mexican schoolchildren, in addition to analyzing the epidemiological implications of this correlation within a global context of children’s oral health. The FPMs, which erupt early in the mixed dentition stage, present a greater window of exposure to the oral acidic environment and other risk factors for caries, significantly increasing their susceptibility compared to other permanent teeth [22,23]. The observed results indicating a strong correlation between the overall DMFT index and the DMFT-FPMs not only indicate a close association between overall oral health and the condition of these teeth but also reinforce the hypothesis that the FPMs act as a "sentinel indicator" of caries experience and prevalence in the pediatric population. In fact, it has been consistently demonstrated that FPMs disproportionately contribute to the overall caries experience in children, exceeding their proportional contribution to the entire dentition as a whole [27,36,37]. This also underscores the importance of first permanent molars in the comprehensive assessment of children’s oral health and in planning effective public health interventions. This finding supports the existing evidence regarding the need to consider first permanent molars as a determining and priority factor in prevention and treatment strategies, both at the individual and collective levels; specific preventive interventions for caries prevention in first permanent molars include the placement of pit and fissure sealants [38-41] and the use of fluoride varnish [40-42]. Therefore, understanding this correlation is vital for the development of public health strategies and the optimization of resources in caries prevention and treatment programs aimed at pediatric populations [38,39,43,44].

The relevance of using both the overall DMFT index and the DMFT for the first permanent molars can be analyzed in terms of effectiveness and precision in detecting caries, suggesting the potential utility of implementing specific measurements for the first molars in population studies, as they may simplify diagnosis and improve early identification of caries risk. The increase in caries experience and prevalence with age [2,10,11,18,23,26] may indicate an accumulation of damage to the first permanent molars, an association observed in various studies, likely due to insufficient access to preventive care and the cost of dental treatment, as noted in other epidemiological studies conducted in Mexico [45,46]. The absence of differences by sex reinforces the idea that caries affects boys and girls similarly, which may guide preventive interventions that do not require gender-differentiated approaches at this stage.

This study found a 10% underestimation of the overall DMFT index when using the DMFT of the FPM as an indicator, similar to that reported in a study conducted on Tunisian children, which reported an underestimation of 12.9% [47]. In another study [27] conducted on Mexican adolescents, the underestimation of caries prevalence (DMFT > 0) was 5.4% (48.6% vs 43.2%), while the DMFT index was underestimated by 0.34 (1.15 vs 0.81), which represents 11.1% for the prevalence and almost 30%, for the average overall DMFT index, of underestimation. This level of underestimation requires a cautious approach when using FPMs as sole indicators in large-scale epidemiological investigations on the caries experience.

Implications in oral epidemiology

Developing oral health strategies that allow us to more efficiently collect caries data from large numbers of individuals for population-based studies will be key to saving costs and reducing the workload of staff performing fieldwork. Epidemiological studies are considered an essential component of public dental health, as they provide realistic data on the prevalence and distribution of oral diseases and are used as a basis for determining and assessing treatment needs in the population. Information from these studies is critical for planning, implementing, and evaluating disease prevention and control program activities, as well as organizing dental care services. Epidemiological indices can ensure that oral health studies are conducted in homogeneous settings, producing valid data that are feasible to collect, repeatable, valid, and reliable in any context or location [27,48].

The high correlation between epidemiological caries indices indicates that the DMFT (decayed, missing, and filled teeth) index of first permanent molars may serve as a simplified surrogate for the use of other indicators in epidemiological studies. This will allow the identification of caries patterns with a reduced need for extensive examination, thus allowing optimal allocation of resources in large-scale studies. Research in communities with lower access to dental services can be conducted by analyzing the DMFT of the first permanent molar. This could help ensure more population-representative caries data in underserved areas while shortening study duration and reducing costs.

The need for specific and effective proposals in caries studies should be emphasized so that there are more specific and adapted public health interventions for the child population. Likewise, this research provides strong evidence that the first permanent molars are key markers of the total caries experience in Mexican school-aged children. Monitoring these teeth may influence the improvement of oral health status and targeted interventions. Further research, especially long-term studies on larger and more representative user groups, is needed to support these findings. These findings indicate that the first permanent molars can be included in surveillance programs developed in school and community clinics. Systematic monitoring of the first molars, which at an early age are really prone to caries, could facilitate the adoption of earlier preventive and therapeutic measures. These findings may improve policies that prioritize the first permanent molars in children's oral health programs. Preservation of these teeth before the age of 15 can reduce the burden of oral diseases and improve the quality of life from an early age, so it will be beneficial for people at early stages.

There are limitations that must be taken into account for the correct interpretation of the results, which are explained below. The cross-sectional design limits the evaluation of the indices; therefore, a longitudinal study would involve following the same group of children over a number of years, periodically assessing their oral health, allowing for follow-up of caries development in the first permanent molars on an individual basis and assessing the impact of DMFT on FPMs, accurately measuring the cumulative impact of first permanent molar caries on the overall DMFT index over time and not just in a single measurement. The number of children analyzed by the study is relatively small and therefore cannot be generalized to all Mexican children. In addition, since the sample was made up of children attending the university clinic, they may not be representative of other regions/socioeconomic classes in the country. The data collection for this study was done from dental records, so the validity of the current analysis depends on the quality and consistency of these records. In addition, it restricts the control that can be exercised over the quality of data obtained from secondary records. The use of secondary data presents the risk of obtaining data that are incomplete, inaccurate, or missing essential information, where variations in recording practices between students or inconsistencies in caries recording may bias the results.

## Conclusions

In conclusion, there is a very strong correlation between the overall DMFT index and the DMFT calculated exclusively for the first permanent molars (DMFT-FPMs) in this sample of Mexican children. This suggests that first permanent molars significantly contribute to the overall caries experience in this population and could be a key indicator in oral health diagnosis and monitoring, considering the percentage of underestimation present.

Additionally, it was found that the prevalence and experience of caries increase with age, but no significant differences were observed by sex. These findings underscore the importance of focusing on the early prevention and treatment of caries in first molars to reduce the global burden of caries in childhood and improve oral health during this crucial stage of development.
